# Lysine-specific demethylase 1 promotes tumorigenesis and predicts prognosis in gallbladder cancer

**DOI:** 10.18632/oncotarget.5279

**Published:** 2015-10-09

**Authors:** Shixian Lian, Yebo Shao, Houbao Liu, Junyi He, Weiqi Lu, Yong Zhang, Ying Jiang, Jun Zhu

**Affiliations:** ^1^ Department of General Surgery, Zhongshan Hospital, Fudan University, Shanghai 200032, China

**Keywords:** lysine-specific demethylase 1, C-myc, invasion and migration and proliferation, prognosis, gallbladder cancer

## Abstract

Gallbladder Cancer (GBC), characterized by invasive growth and infiltrative dissemination, is difficult to diagnose and has poor prognosis. Emerging evidence demonstrates that Lysine-Specific Demethylase 1 (LSD1) has important roles in carcinogenesis, proliferation and metastasis. We studied the roles and molecular mechanisms of LSD1 in GBC. We examined LSD1 expression in 109 paired samples of GBC and normal gallbladder tissues. We found GBC tissues had upregulated LSD1 compared with normal gallbladder tissues (*P* = 0.003), and its high expression was associated with tumor-node-metastasis stage (*P* < 0.0001), Nevin's stage (*P* = 0.0093) and distant metastases (*P* = 0.0070). We found positive correlations between LSD1 expression and other proteins: epithelial–mesenchymal transition markers, C-myc and cyclin-related proteins. Inhibiting LSD1 expression *in vitro* impaired the proliferation and invasiveness of GBC cells and also downregulated c-myc expression and consequently inhibited GBC cell proliferation. LSD1 overexpression promotes GBC development and may be a predictor for a worsened prognosis. LSD1 may be a novel therapeutic target and prognostic tool for gallbladder cancer.

## INTRODUCTION

As the fifth most common biliary tract malignancy, Gallbladder Cancer (GBC) has always been associated with high mortality and poor prognosis worldwide [1, 2]. Owing to its non-specific symptoms, even with the current availability of numerous diagnostic tests, such as computed tomography (CT), magnetic resonance imaging (MRI), contrast-enhanced ultrasound (CEUS), endoscopic ultrasound (EUS) and positron emission tomography (PET), GBC is often diagnosed at an advanced stage when the tumor is non-resectable or metastatic [3, 4]. In most countries the prognosis of GBC is extremely poor, with a 5-year survival rate of 5%–10% and median survival of only 3–6 months from the time of diagnosis [5]. This phenomenon is largely due to the anatomical position of the gallbladder and the high proportion of tumors that are advanced at the time of presentation. Therefore, the identification of relative genes for effective targeting of GBC is urgently needed.

The C-myc proto-oncogene, which was first detected in Burkitt's lymphoma, is over-expressed in most tumors [6, 7], including oral squamous cell carcinoma (OSCC) [8]. As an oncogenic transcription factor, the C-myc protein recognizes the E-box recognition site and several related, non-canonical sequences in the promoter regions of target genes [9]. These targets affect a wide array of biological functions in different cellular models, such as cell differentiation and metastasis. C-myc also plays an important role in normal stem cell biology and may promote the formation of cancer stem cells [10, 11]. In this study, we found that C-myc has a tight relationship with Lysine-Specific Demethylase 1 (LSD1), which is located downstream in the gene sequence.

LSD1 is a flavin-dependent monoamine oxidoreductase and a histone demethylase that serves as an epigenetic co-regulator of transcription [12]. In previous studies, overexpression of LSD-1 was seen in many kinds of human tumors, such as neuroblastoma, lung, colorectal, bladder and prostate cancer [13–16]. Some studies have also demonstrated that LSD1 plays important roles in epithelial-mesenchymal transition (EMT), which leads to repression of E-cadherin, loss of cell adhesion, and increased cell mobility [17, 18]. LSD1 overexpression in GBC cells can drive cell proliferation and enhance cell invasion and migration, increasing tumor malignancy. In contrast, inhibition of LSD1 in GBC cells led to a sharp decline in invasiveness, proliferation and metastatic ability and an increase in apoptosis. Improving patient survival requires an increased understanding of tumor metastasis to allow for early disease detection and development of targeted therapies. Our study aimed to investigate the role and molecular mechanisms of LSD1 in the proliferation and metastasis of GBC.

## RESULTS

### Up-regulation of LSD1 in GBC tissues

**Figure 1:**
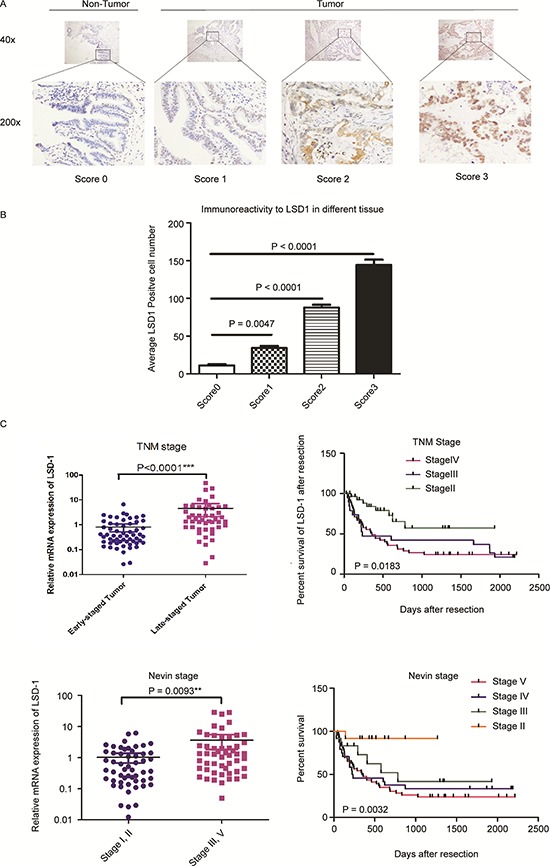
LSD1 expression correlates with GBC progression Positive LSD1 expression by immunohistochemical analysis in normal control specimens (17%) and in GBC specimens (89%). **A.** Representative photomicrographs indicating LSD1 staining intensity scores. **B.** Comparison of LDS1 staining intensity scores among LSD1 positive cells. **C.** LSD1 mRNA expression and percent survival according to Nevin's stage and TNM stage. *P* value was significant at less than 0.05 by Student's *t*-test.

To determine the association of LSD1 with the clinical pathological characteristics of patients with GBC, we examined and compared expression levels of LSD1 in GBC tissues, precancerous lesions and paired normal tissues. Normal gallbladder cells demonstrated slightly positive LSD1 expression, whereas precancerous lesions had increased LSD1 expression. In normal and precancerous tissues, 12.3% and 72.9% of the cells, respectively, were LSD1 positive. Nuclear LSD1 was observed in 75.4% of LSD1-positive GBC, of which, 43.7% of LSD1-positive cells were associated with more intense staining, indicating that LSD1 was expressed at a relatively higher level in GBC relative to normal or precancerous tissues. The results show (Fig. 1A and 1B, *P* < 0.05) an upward trend of LSD1 expression corresponding with increased malignancy of human tissue, with the highest expression being a score3.

To determine whether LSD1 levels are related to GBC progression, we analyzed the association between LSD1 and clinicopathologic status in GBC patients of 109 paired cDNA samples (cDNA samples prepared from GBC patients). As shown in Fig. 1C and Supplementary Table 1, statistical analysis represents a strong correlation between LSD1 expression and tumor differentiation (*P* = 0.0093), tumor size (*P* = 0.0023), lymph node status (*P* = 0.0015), distant metastases (*P* = 0.0070), Nevin's stage (*P* = 0.0032) and TNM stage (*P* = 0.0183). However, LSD1 expression was not correlated with any of the following clinicopathological characteristics: sex, age, differentiation grade and lymphatic invasion (*P* > 0.05) (Supplementary Table 1).

To validate our findings of increased LSD1 levels, we analyzed its expression in TNM (*P* < 0.0001) and Nevin's stage (*P* = 0.0093) patient's tissues. Collectively, these findings strongly suggest that LSD1 expression is correlated with GBC progression and is up-regulated in GBC.

### Baseline characteristics and clinical outcomes of patients

**Figure 2:**
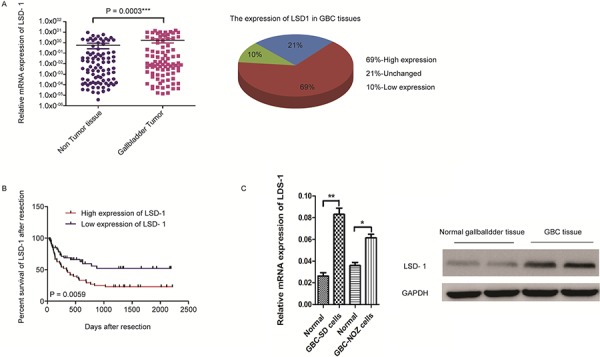
Baseline characteristics and overall survival of patients in 109 paired GBC tissues **A.** LSD1 expression from 109 paired human GBC tissues and mRNA measurement (expression of mRNA as log10 raw counts in the y-axis) plotted across normal tissue and GBC tissue (*x*-axis). **B.** Percent survival of GBC patients after resection based on LSD1 expression. LSD1 overexpression transcripts shows shorter overall survival in GBC (*p* = 0.0003 and *p* = 0.0059). **C.** Western blot analysis and qRT-PCR analysis of LSD1 in normal gallbladder tissue and GBC tissue. The level of the GAPDH gene was used for normalization in the Western blot analysis. *P* value was significant at less than 0.05 by student's *t*-test. ** *P* < 0.01, * *P* < 0.05.

Using quantitative real-time PCR (qRT-PCR), we found that LSD1 gene expression was higher in 109 paired GBC tissues compared to non-tumor tissues (Fig. 2A). Meanwhile, we found that patients with GBC and lower LSD1 expression had better overall survival after surgery, suggesting that LSD1 expression is associated with poor prognosis in patients with GBC (Fig. 2B). Results of qRT-PCR and Western blot (Fig. 2C) showed that LSD1 had a higher expression in GBC than in normal tissues or cells. These results suggest that LSD1 is up-regulated in GBC tissues and is associated with prognosis.

### LSD1 plays a critical role in invasion, migration and proliferation of GBC cell lines

**Figure 3:**
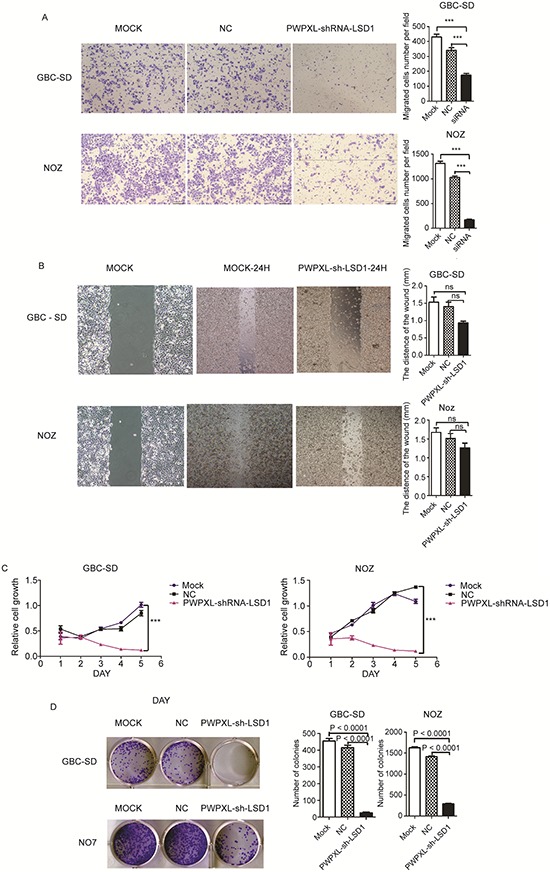
Knock-down of LSD1 inhibit the invasion and metastasis in GBC cell lines Comparison among the MOCK (untransfected), NC (transfected with the PWPXL-GFP plasmid) and LSD1 knockdown/RNAi (transfected with the PWPXL-sh-LSD1 plasmid) groups of GBC-SD and NOZ cells on **A.** Transwell assay, **B.** wound-healing assay, **C.** proliferation assay using CCK-8 and **D.** plate colony formation assay. *P* value was significant at less than 0.05.

To examine the role of LSD1 in GBC cell lines, we performed a wound-healing assay, transwell assay, WST-8 Cell Counting Kit (CCK-8) assay and a colony formation assay. We inhibited LSD1 expression with short hairpin RNA (PWPXL-shRNA-LSD1) in the gallbladder carcinoma cell lines, GBC-SD and NOZ. Invasion and migration was largely reduced compared with that of cells in the negative control group (Figs. 3A and 3B). In the transwell invasion assay, ECM gel was used to mimic the extracellular matrix surrounding GBC cells or the *in vitro* tumor microenvironment. Following the incubation of GBC cells in a transwell chamber for 24 h, there were a greater number of cells that crossed membranes in the control group (MOCK and negative control) than that in the LSD1 knockdown group (Fig. 3A). The average number of invasive cells that crossed the membrane in the untreated control group was more than the RNAi group (*P* < 0.0001).

In the wound-healing assay, cells in the untreated and negative control LSD1 RNAi group exhibited strong migration; therefore, open areas were completely filled and reached saturation within 24 h. In contrast, cells in the LSD1 knockdown group showed a markedly slower migration rate and even an arrested motility. Twenty-four hours later, cells with inhibited LSD1 were still unable to migrate to the open area of the wound and a larger proportion of them died (shown in Fig. 3B). Motility of GBC cells decreased linearly with a decrease in LSD1 expression.

As shown in Fig. 3C, the proliferation of GBC-SD and NOZ cells on CCK-8 assay 48 h after transfection was significantly suppressed after treatment with PWPXL-LSD1-shRNA compared with that of control group cells (*P* < 0.0001). Fig. 3D also suggests the same result on the colony formation assay. Many colonies were formed in the control group compared with the RNAi group. These results illustrate that the downregulation of LSD1 resulted in the inhibition of GBC cell proliferation (*P* < 0.0001).

**Figure 4:**
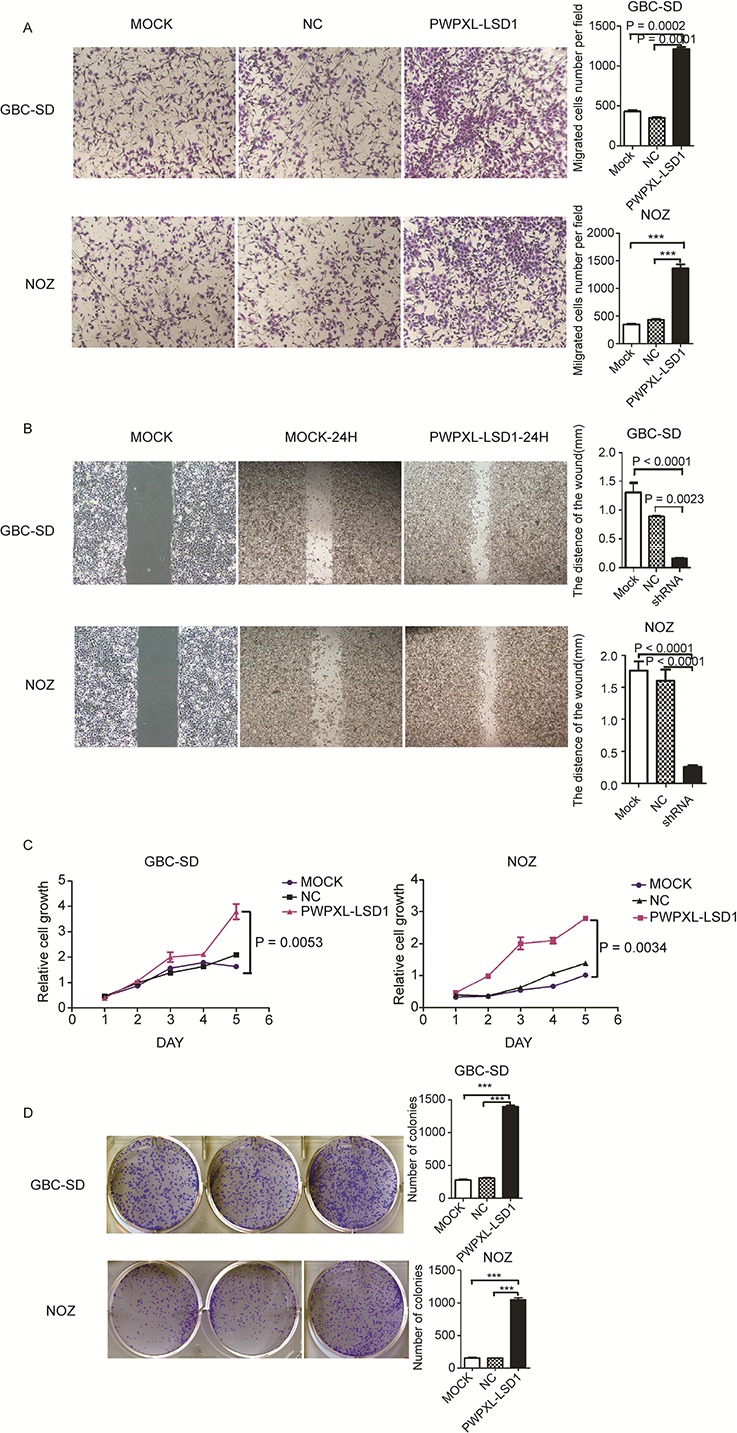
Overexpression of LSD1 promotes the invasion and metastasis in GBC cell lines Comparison among the MOCK (untransfected), NC (transfected with the PWPXL-GFP plasmid) and LSD1 overexpression (transfected with the PWPXL-LSD1 plasmid) groups of GBC-SD and NOZ cells on **A.** Transwell assay, **B.** wound-healing assay, **C.** proliferation assay using CCK-8 and **D.** plate colony formation assay. *P* value was significant at less than 0.05.

After being transfected with a PWPXL-LSD1 plasmid that up-regulates LSD1 expression, the ability to invade, migrate and proliferate was restored in GBC cells (Fig. 4); There were more GBC-SD and NOZ cells that invaded across the chamber compared to that in the MOCK and NC groups (Fig. 4A–4D). Similarly, the ability to proliferate and migrate also increased. From these results, we suggest that LSD1 plays an important role in the proliferation and invasion of GBC cells.

### LSD1 knockdown inhibit EMT in GBC

**Figure 5:**
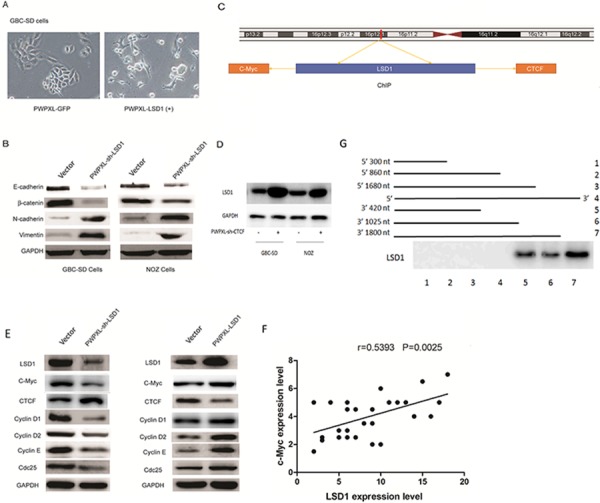
Knockdown of LSD1 inhibits EMT in GBC **A.** Morphology of GBC-SD cells when transfected with PWPXL-GFP and PWPXL-LSD1 GBC-SD plasmids. **B.** Western-blot assay of EMT-related markers in normal control (transfected with the PWPXL-GFP plasmid) and LSD1 overexpression (transfected with the PWPXL-LSD1 plasmid). The level of the GAPDH gene was used for normalization. **C.** Integrated genome viewer for the assessment of LSD1 recruitment to CTCF and C-myc loci based on the publically available UCSC LSD1 ChIPseq dataset. **D.** Knockdown the CTCF, the expression level of LSD1 was increased compared with non-transfected group. **E.** Western blot assay of c-myc, cyclin E, cyclin D2, cyclin D1 and cdc25 in normal control (transfected with the PWPXL-GFP plasmid) and LSD1 overexpression (transfected with the PWPXL-LSD1 plasmid). **F.** The correlation between LSD1 and c-Myc in 29 paired GBC tissues by QRT-PCR **G.** Deletion mapping analyses suggest a 420-nt region at the 3′ end of CTCF that is required for its association with LSD1 to different fragments of CTCF. The level of the GAPDH gene was used for normalization. *P* value was significant at less than 0.05.

The morphology of PWPXL-LSD1 GBC-SD cells was distinct from that of GBC-SD cells transfected with the control plasmid, PWPXL-GFP. While GBC-SD cells transfected with the control plasmid remained tightly attached, showing typical characteristics of epithelial cells, PWPXL-LSD1 GBC-SD cells were more spread out and lost their cell–cell contacts (Fig. 5A). Loss of E-cadherin and upregulation of mesenchymal proteins, such as N-cadherin, are considered biochemical markers of EMT. Loss of E-cadherin is also associated with an aggressive phenotype with poor prognosis. We examined the invasive properties of EMT markers and found that the expression of E-cadherin and β-catenin proteins were downregulated in the PWPXL-sh-LSD1 transfection group (Fig. 5B). In contrast, the PWPXL-sh-LSD1-transfected GBC-SD and NOZ cells had much higher levels of N-cadherin and vimentin protein expression than those of the MOCK control. These results imply that LSD1 positively regulates expression of major EMT-related factors, E-cadherin and β-catenin,and that it may promote EMT progress in malignant GBC cells.

### Downregulation of LSD1 reduces c-myc expression in GBC

As shown in Fig. 5C, CCCTC-binding factor (CTCF), a ubiquitously-expressed eleven zinc finger transcription factor with a sequence-specific DNA binding protein, was discovered in the promoter region of LSD1 genes (http://genome.ucsc.edu/). C-myc was the only gene recognized at the downstream sites of the LSD1 gene in the University of California at Santa Cruz (UCSC) database. This suggests that LSD1 expression may be regulated by CTCF and influences C-myc to alter GBC cell proliferation. To address whether C-myc was directly affected by LSD1 and if LSD1 could be transcriptionally induced by CTCF, we performed deletion mapping analyses [19], identifying a 420-nt region at the 3′ end of CTCF that is required for its association with LSD1 (Fig. 5G). We constructed a PWPXL-sh-CTCF plasmid to knockdown CTCF levels (Supplementary Fig. 1). Knockdown of CTCF caused and increase in expression of LSD1 compared with the non-transfected group. This suggests that CTCF was the up-stream effector of LSD1. To explore the correlation between LSD1 and c-Myc, we used qRT-PCR to analyze the correlation between LSD1 and c-Myc in 29 paired GBC tissues from the original 109 paired tissues. The results show a positive correlation between LSD1 and c-Myc (Fig. 5F). We also tested cell cycle-related proteins (Fig. 5E) to explore other target genes of LSD1. In the PWPXL-sh-LSD1 transfected group, when LSD1 was inhibited in GBC-SD cells, the expression level of C-myc, cyclin E, cyclin D2, cyclin D1 and cdc25 was reduced and the level of CTCF was increased; in PWPXL-LSD1 cells, the reverse was noted. These results suggest that CTCF and c-Myc are up-stream and down-stream regulators of LSD1-mediated proliferation and cell-cycle progression.

### Knockdown of LSD1 expression suppresses the growth of GBC cells derived from tumours in nude mice

**Figure 6:**
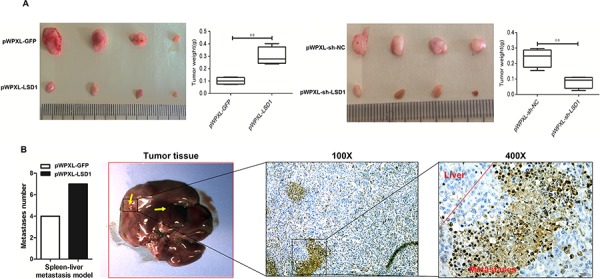
LSD1 regulates tumors proliferation and invasion in GBC xenografts **A.** Gross appearance and size of transplanted tumors after injection of PBS, PWPXL-GFP or PWPXL-sh-LSD1. There was a significant difference between the RNAi (LSD1 knockdown) group and negative control group (NC, LSD1 knockdown negative group). **B.** The number of metastases in PWPXL-LSD1 is significantly more than vector group. The LSD1 expression in tumour cells of different treatment groups using IHC analysis. *P* value was significant at less than 0.05.

To determine the effects of LSD1 on tumorigenesis *in vivo*, PWPXL-shRNA-LSD1 cells, PWPXL-LSD1 cells or PWPXL-GFP as control cells were subcutaneously injected into nude mice. There was a significant inhibitory effect on tumor growth in the LSD1 knockdown group (Fig. 6B). In contrast, rapid tumor growth was observed in the negative control group. Thirty-five days after these observations, the weight of the transplanted tumor was lower in the LSD1 knockdown group than that in the control group. Fig. 6A showed that when LSD1 was overexpressed, the tumor size and weight were more than those of the PWPXL-GFP group. These data suggest that LSD1 suppression inhibited tumor growth in nude mice. We then created a liver tumor metastasis model by injecting the spleen with stable GDC-SD cells (About 30,000,000 cells) transfected with PWPXL-LSD1 to monitor their metastatic ability *in vivo*. Three weeks after inoculation, the total number of metastasis occurrences was much higher in the LSD1 overexpression group compared to the control vector group (Fig. 6C). These results suggest that upregulation of LSD1 promoted tumor cell proliferation and invasion, and downregulation of LSD1 inhibited tumor cell proliferation and invasion. Taken together, these data indicate that LSD1 plays an important role in GBC progression.

## DISCUSSION

Malignant proliferation and metastasis are the two major causes of mortality among patients with GBC. Therefore, it is important for us to understand the mechanisms that help GBC cells acquire invasive/metastatic properties. At present, LSD1 is an emerging target for poorly-differentiated and aggressive solid malignancies.

Using IHC, Western blot and real-time PCR, this study demonstrates that LSD1 was significantly upregulated in GBC. Moreover, among the clinicopathological parameters, LSD1 expression was significantly correlated with lymph node metastasis, tumor differentiation, tumor size, lymph node status, and distant metastases. To the best of our knowledge, there have been no studies that have evaluated the crucial role of LSD1 in the early process of GBC tumorigenesis and as an indicator for prognosis of patients with late-stage GBC by TNM and Nevin's staging.

Similarly, Lv [15] and colleagues found that patients with non-small cell lung cancer and higher LSD1 expression had a poorer prognosis. LSD1 functions as a transcriptional co-regulator for androgen and oestrogen receptors, a component of the nucleosome remodeling and deacetylase (NURD) repressor complex and a regulator of DNA methylation and p53 function [20–22]. Therefore, LSD1 could represent a point of convergence of multiple pro-oncogenic pathways. In this study, we found that LSD1 inhibition has the potential to be a new therapeutic strategy for GBC. Targeted LSD1 inhibition in GBC cell lines showed potent anti-cancer activity both *in vitro* and *in vivo*, with multiple tumor regressions observed in our tumor model. LSD1 plays a key role in many physiological functions, and recent studies suggested that LSD1 knockdown reduced cell growth by affecting expression of several genes involved in proliferation, such as p21 and cyclin A2, and promotes cell cycle arrest [23, 24]. In our study, we found that c-Myc was induced when there was an abnormally high expression of LSD1 by releasing cyclin A-dependent cell cycle arrest *in vitro*.

Given these diverse molecular functions, LSD1 could be of particular relevance to C-myc, and elevated LSD1 expression could identify gallbladder tumors earlier and prevent frequent recurrence. LSD1 may contribute to the constitutive activation of androgen-related growth pathways. However, the exact function of LSD1 in GBC recurrence remains poorly understood. For this reason, we investigated the potential role of LSD1 in promoting EMT progression. Consistent with the prediction based on the UCSC American database, we found that increased LSD1 in human GBC correlated with reduced C-myc in halting the cell cycle. These data illustrate that abnormal LSD1 expression is closely related with CTCF and C-myc; however, this mechanism needs further studies. On the basis of these findings, we identified a statistically significant correlation between increased LSD1 expression and increased CTCF on functional interference of LSD1 function in cultured human GBC cells. However, because the molecular mechanisms of LSD1 in tumor cell invasion, metastasis and proliferation are complex, further *in vitro* and *in vivo* studies will be required to confirm these findings. Nevertheless, these results indicate that LSD1 may be a good target for the treatment of GBC.

In summary, we found LSD1 is significantly upregulated in human GBC tissues. After analyzing clinicopathological parameters, we found that abnormal expression of LSD1 was correlated with poor prognosis in patients with GBC and high levels of LSD1 promoted lymph node metastasis, tumor differentiation, increased tumor size, lymph node status, and distant metastases. Form the results both *in vivo* and *in vitro*, we can safely conclude that LSD1 plays an important role in invasion, migration, proliferation in GBC cell lines. To elucidate the molecular mechanism of this phenomenon, we studied EMT-related proteins and found the transcription factor CTCF, which binds the promoter of LSD1 and promotes the transcription and targeting of C-myc to affect cell function. Our study provides a novel prospect for the development of improved treatment strategies for GBC through the use of the LSD1 protein.

## MATERIALS AND METHODS

### Cell lines, patients and reagents

#### Cell lines

Human GBC cell lines, GBC-SD and NOZ, were purchased from the Chinese Academy of Sciences. Cells were cultured in Dulbecco's modified Eagle's medium (DMEM) supplemented with 10% foetal bovine serum (FBS) (both from Invitrogen Life Technologies, Carlsbad, CA, USA) at 37°C in a 5% CO_2_ incubator.

#### Patients

We enrolled a total of 109 histopathologically-confirmed patients with GBC who underwent primary tumor resection between 2006 and 2008 at Zhongshan Hospital affiliated with the Fudan University (Shanghai, China). All patients did not receive chemotherapy or radiotherapy before surgery. A multi-disciplinary team, including an oncosurgeon, an oncologist and a radiologist, determined the therapeutic regimen. The clinicopathological information and patients’ medical history were documented during post-operative follow-up. Written informed consent was obtained from all patients. The use of the human tissue was approved by the ethics committee of Fudan University.

#### Regents

Antibodies for LSD1, c-myc, cyclin E, cyclin D2, cyclin D1 and cdc25 were purchased from Abcam Biosciences (Cambridge, MA, USA). Antibodies for β-actin, E-cadherin, N-cadherin, β-catenin and vimentin were obtained from Cell Signaling Technology (Danvers, MA, USA). Lipofectamine^®^ 2000 and Lipofectamine^®^ Imax Transfection Reagent were purchased from Invitrogen (CA, USA). Transwell kit for cell invasion assays was purchased from BD Biosciences (Franklin Lakes, NJ, USA); annexin V-EGFP apoptosis detection kit was from BioVision Inc. (Milpitas, CA, USA), and cell counting kit-8 (CCK-8) for proliferation assays was purchased from Dojindo Molecular Technologies Inc. (Kumamoto, Japan).

### Construction of plasmid vectors and transient transfection

Short hairpin RNA (shRNA) that specifically target human LSD1 and CTCF were designed from GenePharma (China). We constructed three shRNAs for LSD1 and CTCF: PWPXL-shRNA-LSD1, PWPXL-shRNA-CTCF plasmid. PWPXL-LSD1, which an overexpression plasmid for LSD1, was also constructed from GenePharma (China). Recombinant vectors were confirmed by the digestion analysis of restriction endonuclease, and all constructed plasmids were confirmed by DNA sequencing. Cell transfection was performed in Opti-MEM (Invitrogen, CA, USA), with the transfection reagent Lipofectamine^®^ 2000 (Invitrogen, CA, USA), following manufacturer's instructions.

### RNA Isolation and quantitative real-time PCR (qRT-PCR)

Total RNA was isolated from GBC-SD and NOZ cells using Trizol (Takara, Japan), according to the manufacturer's protocol. RNA samples were digested with DNase I (Takara, Japan), and then, cDNA synthesis was performed using the reverse transcription kit (Takara, Japan). LSD1 gene expression was evaluated by RT-PCR using SYBR Premix Ex Taq kit (Takara, Japan). Briefly, 3.8 μL of cDNA was amplified in a total volume of 10 μL containing 5.2 μL SYBR Premix Ex Taq and 1 μL of primer. The oligonucleotide primers were synthesized by Life Technology Corporation. The LSD1 oligonucleotide primer sequences were as follows: forward 5′-GTGCAGTACCTCAGCCCAAAG-3′ and reverse 5′-CCGAGCCCAGGGATCAG-3′. The GAPDH primer sequences were as follows: forward 5′-GATGACTACCGTCCACTCC-3′ and reverse 5′-ACTCTGAAAGCCATACCG-3′. At the end of 40 cycles, melting curves of products were obtained. The relative mRNA levels of LSD1 were normalized to the levels of the housekeeping gene, GAPDH, and results were calculated using the 2^−ΔΔCt^ method. All samples were measured in triplicate.

### Immunohistochemistry (IHC)

With antigen–antibody interaction, the targeted protein was visualized and detected using diaminobenzidine (DAB) (Dako Corp, Carpentaria, CA). Briefly, 5-μm tissue sections were removed of paraffin by soaking in xylene and were hydrated in a graded series of alcohol. Then, tissue sections were incubated overnight with an antibody against LSD1 (1:400). On the following day, after being incubated with goat anti-rabbit immunoglobulin (E0432, Abcam), staining was performed using the DAB chromogenic agent. Negative control experiments were routinely performed. The percentage of positive cells was determined by counting 500 cells within five high-resolution fields. IHC staining was evaluated using the percentage, which links IHC staining intensity (SI) with the percentage of positive cells (PP). SI was described by reaction scores between 0 and 3 (0 = none, 1 = low, 2 = moderate and 3 = strong). Accordingly, the number of positive cell nuclei was counted and scored between 0 and 3 (0 = none, 1 = ≤ 25%–40%, 2 = 41%–60%, 3 ≥ 60%). All patients were classified into two groups according to the IHC score: low expression level (0–3) and high expression level.

### Western blot

48 hours after transfection, total protein was extracted from GBC-SD and NOZ cells with radioimmunoprecipitation assay (RIPA) lysis buffer. Using electrophoresis with 10% sodium dodecyl sulphate-polyacrylamide gel electrophoresis (SDS-PAGE) gel, proteins were transferred to the polyvinylidene fluoride (PVDF) membrane (Bio-Rad, USA). This membrane was then blocked with skimmed milk overnight at 4°C. After incubation with primary and secondary antibodies, the expression level of immunoreactive proteins was detected by ECL chemiluminescence reagent (GE Healthcare, Saclay, France).

### Wound-healing assay

Using the scratch method, cultured cells were incubated for 24–48 h in six-well plates (Costar, USA) with DMEM plus 10% FBS until with 90%–100% confluence. A 1-mm wide linear scratch was applied across each well to evaluate for cell migration. Subsequently, 2 ml of DMEM medium without FBS was introduced to repress cell proliferation after washing. 48 hours after wound formation, wound width was measured by Cell Profiler™ Cell Image Analysis Software.

### Cell invasion assay

Cell invasiveness was evaluated using a Transwell chamber, which was precoated with 6 μL of matrigel at 4°C overnight and seeded with 5 × 10^4^ cells. Cells with 500 μL of serum-free DMEM medium were added to the upper chamber and DMEM medium with 10% FBS supplement was added to the lower chamber. Cells were incubated for 20–36 h without treatment. The cells on top of the membrane were removed downward and cells that penetrated the membrane were fixed in ethanol, followed by crystal violet staining. The number of cells on the opposite side of the membrane was counted under the microscope in four random fields of vision.

### Cell proliferation assay

Cell proliferation rates were measured on 96-well microplates using the WST-8 Cell Counting Kit. GBC cells were seeded (1 × 10^3^ cells/well) in DMEM medium containing 10% FBS. After overnight incubation, growth rate was determined using the WST reagent on a microplate reader in the 450-nm absorbance spectrum. All samples were measured in triplicate.

### Plate colony formation assay

Single-cell suspension at a density of 5 × 10^2^ cells/mL was seeded in 12-well plates with 1 ml DMEM plus 10% FBS per well. At 12 h after inoculation, cells were checked under a microscope to confirm that single cells were formed for further observation. The cells were then cultured for more than 10 days until visible colonies were formed and then were stained with crystal violet and photographed before counting the colonies. Each assay was performed in triplicate.

### Tumor model in nude mice

Age-matched adult male nude mice, four weeks old, were housed in a temperature- and light-controlled environment with a 14/10-h light/dark cycle. The GBC-SD cells were harvested in serum-free culture medium and the concentration of the cell suspension was adjusted to 5 × 10^7^ viable cells per ml. The cell suspension (0.2 ml) was subcutaneously injected in the right lateral of the mice oxter. The tumor was formed within approximately 18 days after injection. The mice were randomly divided into three groups: 1) the PWPXL-LSD1 group, where the mice were transplanted with the stable over-expression of LSD1 cells; 2) the PWPXL-GFP as the negative group, where the mice were transplanted with cells stable transfected with the PWPXL-GFP plasmid; 3) the PWPXL-sh-NC group, where the mice were transplanted with cells transfected with the PWPXL-sh-NC plasmid as a negative control; and 4) the PWPXL-sh-LSD1 group, where the mice were transplanted with cells stable transfected with the PWPXL-sh-LSD1 plasmid to knockdown the expression level of LSD1.

After 30 days of observation, the mice were sacrificed and an autopsy was immediately performed. The tumor tissues were then fixed in 4% paraformaldehyde.

### Statistical analysis

Quantitative data were expressed as mean ± 95% confidence intervals (CI) using Graphpad Prism 5 (Graphpad Software Company, USA) in figures. One-way analysis of variance and student's *t*-test were performed to determine the relationship between LSD1 expression and GBC clinico-pathological factors, including sex, age, pathological differentiation, infiltration, lymphatic metastasis and overall survival. Kaplan–Meier survival curve was drawn and significance was calculated with the log-rank value. Comparisons were considered statistically significant when the *P* value was less than 0.05.

## SUPPLEMENTARY FIGURE AND TABLE



## Abbreviations

LSD1: Lysine-specific demethylase 1
GBC: gallbladder cancer
CTCF: CCCTC-binding factor
Nevin's stage: The staging of GBC is based on the depth of penetration and extent of spread In 1976, Nevin. Stage I Intramucosal tumor; Stage II Tumor extends to the muscularis; Stage III Tumor extends to the serosa; Stage IV Transmural involvement and cystic lymph node involvement; Stage V Direct extension to the liver and or distant metastasis
TNM stage: T describes the size of the original tumor; N describes regional lymph nodes that are involved; M describes distant metastasis
