# Supplementary material for: Lysine-specific demethylase 1 promotes tumorigenesis and predicts prognosis in gallbladder cancer
Source: Oncotarget. 2015 Oct 9;6(32):33065–76. doi: 10.18632/oncotarget.5279 (PMC4741749; doi:10.18632/oncotarget.5279)
Supplement: Supplementary file 1 [file oncotarget-06-33065-s001.pdf]

SUPPLEMENTARY TABLE AND FIGURE

Supplementary Table S1: Relationship between LSD1 expression and clinical pathologic factors of patients with Gallbladder cancer

Parameter	No. of patients	LSD-1 (low)	LSD-1 (high)	*P*-value
Sex				0.4599
male	30	11	19	
female	79	21	58	
Age				0.0760
<60	28	16	12	
≥60	79	20	59	
Pathological type				<0.0001
Score 0	23	23	0	
Score 1–2	49	6	43	
Score 3	37	3	34	
Tumor differentiation				0.0093
I	6	1	5	
II	33	13	20	
III	70	21	49	
Tumor size (cm)				0.0023
≤5	32	14	18	
>5	77	9	68	
Differentiation grade				0.0647
Well-moderate	57	24	33	
Poor-undifferentiation	52	30	22	
T stage				<0.0001
T1-T3	62	21	41	
T4	46	6	40	
Lymph node status				0.0015
Negative	63	36	27	
Positive	46	7	39	
Distant metastasis				0.0070
M0	37	22	15	
M1	72	14	58	
TNM stage				0.0183
I-II	49	16	33	
III-IV	60	14	46	

(*Continued*)

**Parameter**	**No. of patients**	**LSD-1 (low)**	**LSD-1 (high)**	***P*-value**
Lymphatic invasion				0.3072
Negative	58	30	28	
Positive	51	12	39	
Nevin's stage				0.0032
I-II	42	27	15	
cIII-IV	67	10	57	

$^{*}P < 0.05$

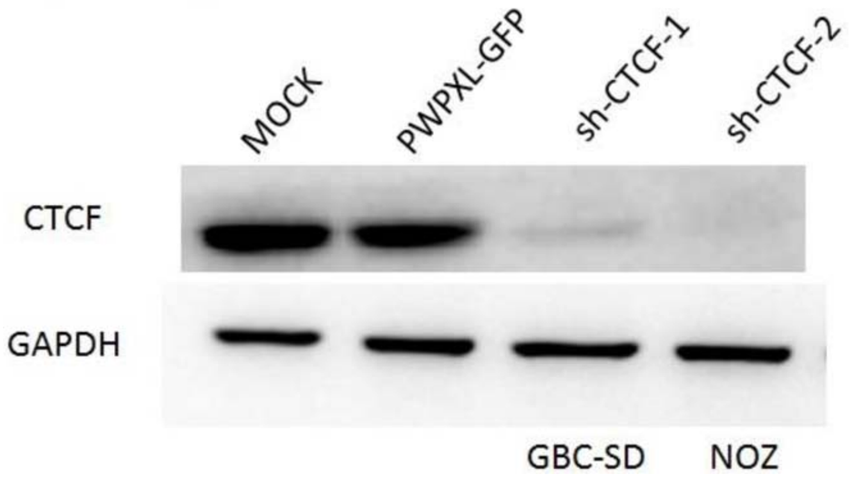

Supplementary Figure S1: The interference efficiency of PWPXL-sh-CTCF in GBC-SD and NOZ cells. Western blot assay of interference efficiency of CTCF in GBC-SD and NOZ cells. The expression of CTCF in the group which transfected with the PWPXL-sh-CTCF plasmid, was significantly lower than the expression level in MOCK and control group (transfected with the PWPXL-GFP plasmid).
